# Nurturing Socioculturally and Medically Appropriate Palliative Care Delivery: Lessons Learned by Israeli Medical Faculty

**DOI:** 10.1007/s10943-022-01522-8

**Published:** 2022-03-09

**Authors:** Piret Paal, Anne Müller, Woukelyne Gil, Gil Goldzweig, Frank Elsner

**Affiliations:** 1 Institute of Nursing Science and Practice, Paracelsus Medical University, Strubergasse 21, 5020 Salzburg, Austria; 2 Department of Palliative Medicine, RWTH Aachen University, Aachen, Germany; 3 Department for Internal Medicine, St. Elisabeth-Krankenhaus Geilenkirchen, Geilenkirchen, Germany; 4 Rambam Health Care Campus, Tel-Aviv, Israel; 5 School of Behavioral Sciences, Academic College of Tel-Aviv-Yafo, Tel-Aviv, Israel

**Keywords:** Palliative care, Culture, Religion, Access, Education, Spirituality

## Abstract

Israel is one of the few countries worldwide with a national policy and defined standards of palliative care (PC); its culture is highly diverse and more traditionally oriented in comparison with Western countries. This study describes the current state of PC in Israel through examination of: (1) its current status, self-image and structural factors; (2) its relation to cultural and political characteristics; and (3) the chances, goals and obstacles of advancing PC in Israel. Face-to-face interviews were conducted at all five public medical faculties in Israel from November 2017 to February 2018. The following findings are reported: (1) definition of palliative care, (2) multidisciplinary approach, (3) special role of nurses, (4) personal perceptions of death, (5) understanding the role of medicine, (6) specialty palliative medicine, (7) religious, spiritual and cultural aspects, (8) political and economic aspects, (9) obstacles and weaknesses, and (10) prospects and goals of palliative care. Participants perceive PC as an integrative healthcare service that should be available to all patients, including children and their families, at any stage of illness. They internalize that PC principles apply regardless of ethnic, cultural, and religious background. Utilizing nurses’ leadership, enhancing multidisciplinary teamwork, and person-centered approach, supports better PC to more people.

## Introduction

Palliative care (PC) is a fundamental component of national health systems and an essential service within Universal Health Coverage reforms. It is highly effective at relieving the pain and suffering of people living with and affected by life-limiting illness, optimizing their quality of life from early in the course of their disease until the end of their lives (Morris & Davies, 2020). The shift from hospice and end-of-life (EoL) care to integrated PC services has affected the global PC policy-making and makes it difficult for governments, service providers and faculty to keep up. Integrated PC contains a strong socioeconomical side (Scott, 1994). The Human Rights Watch closely monitors access to PC and pain relief. They report that the majority of governments have been reluctant to invest in high-quality PC. In 2018, a minority of countries (37%) had a national policy that includes PC. PC is least likely to have funding available compared with other core services. There is a large country-income gradient for PC funding, oral morphine availability and integration of PC services at the primary levels of the health system (Sharkey et al., 2018). In 2019, funding for PC was available in 68% of countries, about 14% of people who need PC actually get it, and only 40% of countries reported that the services reached at least half of patients in need*. *Governments’ reluctance to invest in PC services, research and education is a global challenge (Clark et al., 2020). Therefore, monitoring country-specific advantages and barriers is of utmost importance.

Israel has a long history of PC that can be traced to Jewish, Christian and Muslim traditions of hospices as long as to religious institutions supplying and providing for the needy, sick and dying pilgrims. The names of some of these institutions are still remembered through the names of old buildings in Jerusalem (Waller, 1997). The oldest *hospes*—a place of hospitality for the sick and dying, for travelers and pilgrims—were situated in the region that introduced the modern hospice movement during the late 1970s. Specially trained cancer nurses established the first outpatient PC service (Silbermann et al., 2012). The first hospice opened in 1983 (Emanuel & Cherney, 2012). History, tradition, and the sociocultural structure of Israel have made culture and religion an integral part of PC in Israel. Nevertheless, this could have come at the expense of spirituality. The European White Paper on spiritual care education in palliative care points out that the evidence on culturally specific needs in palliative care is limited. Patients prefer services that make them feel safe and well-served. Healthcare professionals need to protect patients from harmful value-based beliefs and practices (Best et al. 2020). Assessment of spiritual, cultural and religious beliefs is essential; however, it is absent and poorly utilized (Finn et al., 2020).

The policy reforms to make PC a multidisciplinary service are recent. In 2005, the Israeli Ministry of Health (MoH) established a commission to determine what is needed to integrate PC into the healthcare system. This commission was to help the Ministry develop a national policy and define the standards of care. Simultaneously the Israeli parliament enacted the *Dying Patient Law* to regulate decision-making at the EoL. The law addresses a living will to ensure patient autonomy and sanctity of life, a culturally relevant topic in Israel. The demand to offer PC to all patients with life-limiting illnesses and their families is also reiterated and emphasized as a right of all Israeli citizens (Steinberg & Sprung, 2007). Israeli National PC Policy (INPCP) was published in 2009 (Ben Ami & Yaffe, 2015). In 2009, PC was included in the “health basket” of Health Maintenance Organization (HMO) public insurance services. Accordingly, all Israeli citizens are entitled to free PC when needed (Emanuel & Cherney, 2012, The State of Israel: Ministry of Health, 2009). The policy from 2009 stated that by 2013 all healthcare facilities must provide palliative care of the highest quality. The policy also defined the minimum standards, scope and tasks of a PC team. The implementation of legislative resolutions began approximately ten years after their approval (Rosen et al., 2015). In 2015, an inventory and quality review of the goals from INPCP of 2009 was carried out. Many hospitals and geriatric institutions had established PC teams to meet the policy criteria. The inventory revealed that many teams were understaffed and insufficiently trained and the quality of care was compromised (Shaulov et al., 2019).

In 2016, a new national plan to enhance PC was introduced. The development of this National PC Program (NPCP) involved the MoH, the Ministry of Welfare, health insurance companies, universities and elected officials. The first national meetings of PC specialists to advance PC have taken place. The implementation of the resolutions of this plan is ongoing (Fischer-Reif et al., 2016; Hadad et al., 2013; Shaulov et al., 2019).

Israel can serve as a case study for understanding the obstacles and challenges in developing PC services and training. First, Israel has a very good public healthcare system and holds a high rank among the OECD countries (Hadad et al., 2013). Nevertheless, PC services are provided mostly to cancer patients (Shaulov et al., 2019). Exploring obstacles to PC implementation may help to fill the gap and, furthermore, improve the country’s overall healthcare system quality (Elsner et al., 2021). Second, Israel is a highly diverse country. On the one hand, Israel belongs to upper income countries with high living standard, access to education and low infant mortality rates (Beider & Goldzweig, 2016). On the other hand, the Israeli society remains very traditional, with higher number of children per family, lower age at first marriage, etc. The Israeli society can be described as an amalgamation of cultures, religions, and people from different origins including the Jewish immigrants from many countries, Muslim Arabs, Christian Arabs, Druze and Bedouin. Understanding PC with this background of westernized healthcare and a tapestry of cultures may contribute to improving of PC in other exceedingly diverse places in the world.

## Aim

This article reports on medical faculty’s perceptions of the current state of the art in PC in Israel. The article addresses the following research questions:

- What are the current status, self-image and structural factors of PC in Israel?
- How do cultural and political characteristics influence PC in Israel?
- What are the chances, goals and obstacles of advancing PC in Israel?

## Methods

This is a qualitative explorative study using semi-structured interviews (DeJonckheere & Vaughn, 2019) to develop new knowledge of medical faculty’s experiences on PC. The interview guide (Appendix 1) was developed in advance and validated via test interviews. Face-to-face interviews were conducted at all five medical faculties in Israel from November 2017 to February 2018.

All participants (*n* = 11) signed an Informed Content Sheet and agreed to the audio recording. The ethics committee of the RWTH Aachen University has issued a Letter of No Objection for this study (EK 182-17).

F4 transcript software was used to prepare the Verbatim. All interviews were analyzed anonymously using code names. The inductive text analysis method was applied, to generate the statements and results based on the collected data (Kuper et al., 2008; Mayring, 2015). Five transcripts were used to create a preliminary category system. The analysis was conducted in the following sub-steps: (Z1) Paraphrasing; (Z2) Generalization to the level of abstraction; (Z3) First reduction by one investigator (Table 1); and (Z4) Second reduction, where all authors collaborated until categories were finalized (Vaismoradi et al., 2013).

**Table 1:** The sub-steps textual analysis according to Mayring (14)

Interview	Row	Paraphrase (Z1)	Generalization (Z2)	Reduction 1 (Z3)
EH63B07	7	PC is a type of management	Definition PC: Management	Definition PC Management
EH63B07	7–9	PC is a way of caring for patients and their families in terms of physical, psychological and social suffering	Definition PC: For patients and families. Reference to physical, psychological and social suffering	Includes families Focus on physical, psychological, social and spiritual suffering From diagnosis throughout the course of the illness From diagnosis throughout the course of the illness
EH63B07	11–13	Also spiritual needs are taken care of	Definition PC: Reference to spiritual needs	Main focus on symptom management
EH63B07	18–20	One needs PC from the moment of diagnosis throughout the course of the illness	Definition PC: Required from the time of diagnosis throughout the course of the disease	Difference to other disciplines All symptoms treated Patient at the heart
EH63B07	20–24	Children need much PC when they have cancer, even if the prognosis is usually good	Definition PC: also required in case of good prognosis (children)	
EH63B07	38–40	The difference to other disciplines: In addition to physical symptoms, we also take care of psychological, social and spiritual ones	Difference from other disciplines: only care about physical symptoms	
EH63B07	40–43	In a hospital, the focus is usually on the disease, not on the patient. This is the main difference	Main difference from other disciplines: disease instead of patient at the heart	

## Results

**Fig. 1:**
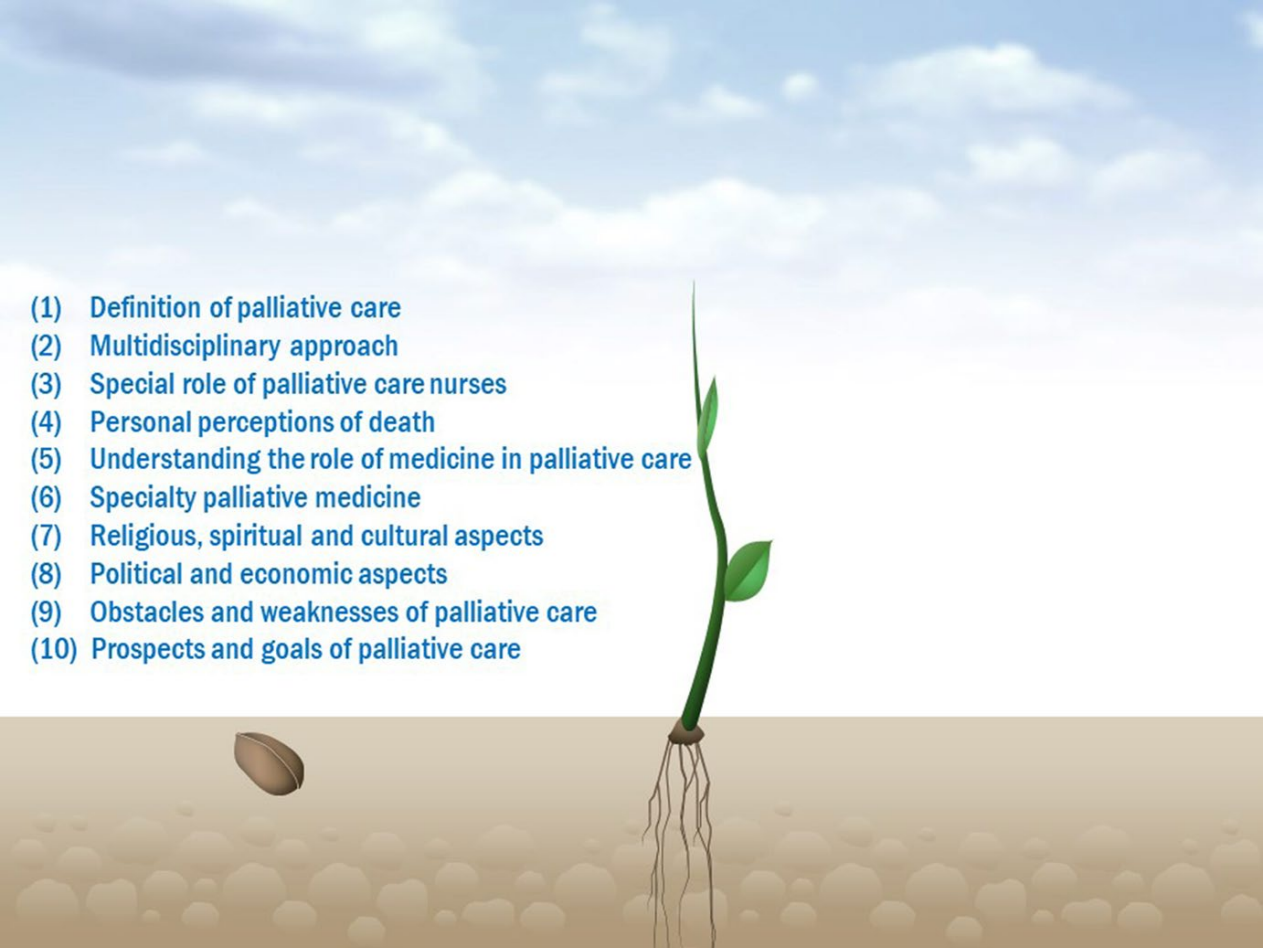
The list of themes

The qualitative analysis of the interviews resulted in a coding tree consisting of 1838 codes. The ten final categories are presented in Fig. 1. The following text reflects a summary of the interviewees’ thoughts and ideas.

### Qualitative Interviews: Demographic Data

We conducted three interviews in The Hebrew University, and two each in Ben-Gurion University, Bar-Ilan University, The Technion, Tel Aviv University. All in all, eleven faculty members (ten physicians and one social worker) participated in this study. All, but one participant, had frequent contact with patients receiving PC. Seven worked in PC wards. There were four PC specialists among the respondents. Six had completed a fellowship or specialist training in PC abroad. All interviewed faculty members were involved in PC teaching to undergraduate medical students or involved in training during clinical internships.

### PC in Israel: Results of the Qualitative Inquiry

#### Definition of PC

The interviewees mentioned various special features of PC as criteria connected with the definition of PC. The most frequently mentioned defining criterion was the holistic nature of PC, which means addressing and treating physical, psychological, spiritual and social suffering. It is essential to be informed about patient’s expectations and needs to define patient-centered treatment goals. Overall, the focus of treatment is on the patient’s quality of life and symptom management. It is important not to accelerate death, but also not to delay it. The treating physicians should help their patients make the best out of life and help them deal with life and death questions. PC should support the families, but the day-to-day running of the ward is not family-oriented. Death is unpredictable; therefore, PC should start at the time of diagnosis and follow through the entire course of the disease.

*So personally, as a physician, I see patients with fibromyalgia and I see patients with metastatic cancer and I see patients with ALS and I see patients, I mean as a PC physician I see all of them. So, for me, it's the attitude of managing the quality of life of any patient that deals with any illness. It's a very broad (...) way of using PC.* (A5T11, par. 4)

### Multidisciplinary Approach

The interviewees emphasized that PC is a multi- and interdisciplinary field in which the hierarchies are flat and the team members support each other. In contrast to PC and perhaps the general medicine, where the patients are at the heart of care, other specialties much more treat diseases. They are less oriented toward the individual, holistic treatment goals and quality of life. Other disciplines should be more oriented toward the principles of PC including teamwork and support for families. Teamwork and mutual encouragement within the team are particularly essential for patients with complex health-related suffering. The multidisciplinary team should consist of physicians, general nurses, specialized PC nurses, social workers, psychologists and spiritual caregivers. Psychotherapists and dieticians were also listed as important. Some PC units in Israel have music therapists, masseurs, beauticians, medical clowns, and specialists in traditional Chinese medicine (acupuncture). The roles within the team complement each other and regularly meetings help to improve the daily patient care as well as PC competencies within the team.

Since the patient clientele in Israel is multinational, diversity management in teams is essential to overcome linguistic barriers and to understand religious and cultural needs of people from various nationalities, ethnic backgrounds and religious groups.

### Special Role of PC Nurses

There are very few doctors in Israel who work exclusively in PC. Hence, nurses with specialist PC training are of special importance in Israel. These nurses are particularly active in outpatient PC or employed in hospitals and hospices. Some PC nurses have their small clinics. Some offer consultations across the hospital. Except for a few procedures and signature authority, PC nurses have almost the same tasks as doctors. They examine patients, plan care, coordinate examinations and, with some restrictions, adjust medication. The nurses are responsible for coordinating patients with complex care needs and for follow-up. In outpatient PC, they check the home for patient equity and medication compliance and coordinate the palliative team. PC nurses are active in research and in teaching:

*PC (.) in Israel, for many years, was mainly provided by very few doctors. And many more (.) nurses, oncology nurses, some oncology nurses with PC training. Now there is special PC/ There are palliative nurse specialists. Every hospital in Israel has a PC Nurse. (.) They do most of the PC provision in Israel, as far as I'm concerned. They have a major role in PC development and provision of PC in Israel. The nurses. It is they who moved the system. And there were many more PC Nurses than there were PC doctors. (M5S05, par. 42)*

### Personal Perceptions of Death

Dying is part of life, life is finite, and death is inevitable. Peaceful dying is important for patients and families. Nevertheless, there is more than one definition for what could be considered “a good death.” This is uniquely individual. It is a privilege to accompany people at the end of their lives. It is important to speak openly with patients about death so that they can make autonomous decisions. Even if memories of the deceased sometimes make people sad, these are generally good and heartwarming. It is particularly challenging to experience dying processes in one's circle of acquaintances. Like any other separation, the death of a close one makes causes sadness and grief. In such situations, building walls does not work:

*So, I don't build any walls. Never. It doesn't work. I tried a few times. (makes the sound of a crack) Completely failed. But perhaps there are other people with different personalities who manage to build the wall. And (..) live happily with the walls. I mean you can build a wall, but you want to be happy with yourself. (..) So my feeling is: don't bother to build them. It's a waste of energy. And that energy that you spend building a wall, spend it accepting, understanding, learning. (O6B06, par. 127)*

Participants agreed that personal and professional attitudes toward death and dying have to be in agreement. Personally, w*hen dying, wishes from living the best possible life in a finite time in the world, to be sedated early, or have an aggressive curative therapy were uttered.*

### Understanding the role of Medicine in PC

Medical knowledge and compliance with the law define a good doctor. To be a good doctor and PC practitioner, communication and building relationships are crucial. To help in life and death, to give the best care means that besides the clinical tasks, other requirements have to be filled. For example, to stay by the patient’s side and not leaving during the dying process. Caring about other’s health and love of medical work is imperative. Constant attentiveness toward feelings and self-reflection are essential to improve oneself. Especially in emotionally difficult situations, being aware of the role of a physician is important. Foreign colleagues and training periods abroad inspire to change the practice. The desire to do things differently than problematic medical role models from the past suggest may be a driving force.

### Specialty Palliative Medicine (PM)

PM is a new, just emerging, field of expertise. It is a bottom-up movement: Existing PC teams aspire to spread their practice. Outpatient PC has thus developed from outpatient care, and this is now slowly making its way into hospitals. Such development is different from the USA, where outpatient PC emerged from inpatient care. Most PM in Israel takes place in geriatrics or internal medicine. The standardization of PC is challenging, but it is covered by health insurance and has become a medical specialization. PC is also increasingly promoted top-down, thus, it is developing well:

*And I say that very early on I realized that (..) PC is not going to develop through the initiative of the health provider, because it's expensive and it's not popular. (…) So, it has to come from above and below. (.) So we worked above, and we worked below. And it's working. (O6B06, par. 93-95)*

The field is still very dependent on a few experienced doctors. PC is not a particularly popular specialty, as it does not benefit doctors financially or guarantee recognition among colleagues. However, PC is a field with high professional quality of life. Physicians receive a lot of warmth and acknowledgment from patients. The burnout rate within PC teams is generally low.

### Religious, Spiritual and Cultural Aspects

How culture and religion influence PC in Israel provided lots of controversial discussions. The principles of PC are independent of religion and culture. On the other hand, religion has a significant influence on everything that happens in Israel.

*Breaking Bad News* is a special challenge for doctors in PC. Communication regarding diagnosis and the answers about the prognosis require good communication skills. Interviewees mentioned that most patients want to know their diagnosis. It makes communication easier and helps to build a relationship. The legal requirement is to inform patients and most of the patients have guessed their diagnosis anyhow. Yet, direct communication is often a difficulty in Israel. Patients or relatives do not want to talk openly about their disease. Thus, relatives ask for information before the doctor has the opportunity to address the patient, and then, family members plead, not to inform the patient:

*That's something called the "conspiracy of silence". You know, there's (..) I come into the apartment and the son waiting outside says: "Don't tell my father what he has, because if he knows, he'll commit suicide. He is very sensitive." Well, fine. (coughs) Sit down with the father, we talk, we can talk. After about 20 minutes, he will tell his son: "Joseph, please, go to the kitchen and prepare a cup of coffee for the doctor and take the cake from the upstairs and do this and do that." And as soon as Joseph has gone to the kitchen, the father would say: "I hope you've not told my son about the liver metastases. He will be devastated when he finds out. So, let's keep it between you and me." (…) So, I will say to him: "That's strange, he said the same thing about you." "Really?" "(Laughs) Ask him!" And then Joseph comes back, and dad asks him: "Joseph, do you know about the liver (.)?" And he looks at me and he says: "You told my father?" I said "No, I didn't tell him. He told you." (..) And that happens quite often. (O6B06, par. 63-67)*

Israel is currently changing, which means more openness emerges in such talks. Secular people tend to seek direct communication. Albeit, too direct conversations may lead to refraining from seeing a doctor further on. Some may feel anxious facing direct communication.

It is easier for doctors to talk to the families than to the patients. The common practice is that the attending physicians tend to leave delivering the bad news to the patient to the PC specialist. In complex cases, PC physicians would thus coordinate their work with the team before addressing the patient.

Interviewees refer to the question about the prognosis is referred to by the interviewees as equally challenging. The response strategies would be vague formulations such as “weeks to months” or “months to years,” or, in other cases denying such questions or replying with a counter-question. The latter was somewhat anecdotally explained as deriving from Talmudic teaching to ask a counter-question to every question. The counter-question helps to understand why the patient is interested in the prognosis. This helps to choose a strategy for communication. Many patients are interested in how they would fare in the coming time. Thus, the talk would be about the quality of life and less about numbers. Patients want to hear whether they can experience certain things in their lives, how their everyday life will be and how these specific goals can be achieved: *What was important for her is: How do I plan to do what I have left to do, and I want to do before I go away. (E6B07, par. 48).*

Language barriers are a special feature of doctor–patient communication in Israel. Many doctors are fluent only in Hebrew, which is incomprehensible for many older Arabs and immigrants. A translator is called only for important matters. Family members may function as translator, with the risk that information is getting misinterpreted. Hence, elderly patients, with protective families, are particularly at risk of being excluded of clear and complete communication.

Culture and religion have little or no influence on PC in Israel. The provision of care is the same to all patients. Religious views harmonize well with PC. It was suggested that the reactions of the dying are always the same:

*I was in Europe, the United States, Africa and India and in the end everywhere people ask the same questions and are confronted with the same challenges when facing death. They are all afraid to die. (O6B06 par. 77)*

PC is not about culture and religion. For the best possible medical treatment, a doctor should adapt to the patient’s culture, religious wishes, spiritual inclination, general needs, behavior and habits. It is essential to ask patients openly: what influence spirituality and religion have on their lives, how they deal with death, and their wishes for the dying process. There are various possible answers to these questions. The doctors should respect the faith of their patients:

*It would be wrong to make assumptions for such big groups of people. Within one culture, there are many different cultures included. A culture can be divided into different cities, neighborhoods, families, and finally even individuals. Everybody is different. And the way people are dealing with death is much more determined by human nature than by their culture. (O6B06, par. 80)*

Israel is very heterogeneous in terms of religion and culture. Still, people’s approach to life and death issues is influenced by gender and age differences as well as religious and ethnic background. In most cases, the individual nature of people influences their attitude toward the way they deal with PC more than their specific culture or religion. Religion has more influence on extreme or ultra-religious patients and families in their PC interactions and decisions. The more secular or non-observant tend to be more open, rational and solution-oriented.

Interviewees referred to differences between Jews and Arabs (both Muslim and Christian Arabs) regarding attitudes toward the disease and the prospect of death. According to such observations, Muslims and Arab-Christians are characterized by a higher degree of fatalism: accepting the unalterable fate. In Islam, this may be related to the belief in a continuation of life beyond death. Jewish patients tend to insist on maximum therapy at the end of life. For religious Jews, this insistence is in accord with their faith. For the more liberal and secular, religion is less of a factor, but rather them being traditionally considered “fighters.”

*Israel is very unique in many ways. One of them is, 'cause it's a country that still lives on its idea that it's fighting for its survival. The very idea of PC is a major turn-off (laughs) for a lot of people. [...] People like to feel that they are FIGHTING. And they are FIGHTING. And they are willing to go down FIGHTING. And there is a lot of that talk in the air (laughs). (D3J02, par. 64-66)*

*Interviewees provided several examples where particular religious and cultural traditions or customs influence PC routines. Such are Jewish Rabbis whose advice and views determine how PC is perceived.* In some ultra-Orthodox families, the Rabbis even make medical decisions and give directions on issues at the end of life. When this is the case, it is problematic as patients may refrain from seeking available medical advice or spiritual guidance from trained professionals.

The interviewees stressed that spirituality is a broad term, which includes more than just religion:

*In Israel there is some sensitivity about religious issues. You know, bringing religious issues to, maybe to people who are not so interested in this. So, for some people spirituality is religion. But for others it may be literature, poetry, music... you know? People have different spiritual needs. (O5J01, par. 41)*

Besides working together with broadly educated hospital chaplains who care for patients from all major religions, spiritual assessment is seen as a crucial part of patient-oriented care:

*There is not a single patient that I don't ask about his spiritual beliefs. It's important. What does death or disease, first disease and then we come to death, mean for him? "What does it mean for you to be ill?" Somebody feels it is a punishment, and somebody feels: "You know how do I get this? This is that..."; and somebody thinks, I have a lot of women here who say: "It's my bad marriage. I have a lot of men who say: "It's my work". I want to hear all this. Because if I want to help them, we need to, I need to work with this. (A5T11, par. 83)*

### Political and Economic Aspects

The political situation affects the everyday life of Israelis and influences their lives in many areas. In terms of PC, the influences are compound, albeit PC services are provided to all on an equal basis.

The treatment options for patients from The Gaza Strip are affected by the political situation. Patients from there often come to Israel to receive better medical treatment. In Gaza, hospice and outpatient PC are in a problematic state, the supply of medication is unstable and treatment options are poor. Patients from Gaza receive medical treatment in Israel at a reduced price, paid either by the patients themselves or by their government. The language barrier and a certain degree of distrust are common to these doctor–patient contacts.

Since the military operation 2014, fewer patients from Gaza have been treated in Israeli hospitals. Some patients are refused entry to Israel, and an increased uncertainty exists as to whether patients can reenter the country for further treatment after initial treatment in Israel. The separation and the border controls have complicated gaining rapid access to medical care. Even for doctors from the Palestinian territories, the journey to their workplaces has become complicated.

The interviewees express appreciation of cooperation between Palestinian (both from the Gaza strip and from the West Bank) and Israeli doctors. Both want to advance medicine, and Israelis help to establish PC in the Palestinian territories. There is more cooperation in medicine than in other areas of life.

In Jerusalem, where the Palestinian territories are very close, there are many Palestinian patients and doctors:

*Palestinian doctors are looking after Jewish patients and Jewish patients [sic!], Jewish doctors looking after Palestinian patients. And everyone is treated very very equally. (…) And that is easier with Jerusalem residents” (A5J03, par. 54-56)*

Tensions may occur between different patient groups: *“On the day where there is a terror attack here and I have in a room and Ultraorthodox man and an Arab man from Ramallah, it makes things difficult.” (DH37J02, par. 94).*

The health situation of the asylum seekers in Israel is worrisome. These refugees may purchase health insurance from HMO Meuhedet for 200 New Israeli Sheqel (NIS = 50€). Asylum seekers with children make use of this insurance. However, many such refugees without families are not insured. They receive medical treatment only in case of emergencies. PC is provided to refugees from Syria since there is no outpatient PC in Syrian communities. Refugees with life-limiting illness are flown back home if they wish so. An interviewee said that it is cost-effective for the health insurance provider:

*We had some asylum seekers that were diagnosed with very, life-threatening diseases and there is a way to send them back home, if they want to die at home, to the Philippines, and India. So actually, the hospital would/ Well, it's cost-effective, right? But they will have them fly back. (O4T10, Abs. 96)*

### Obstacles and Weaknesses of PC

Many Israelis have limited knowledge about PC and its objectives. Many are also unaware of their patient rights. Partly, there is a misconception of PC as standing for euthanasia and the shortening of life. An interviewee referred to this misconception as being widespread in the MoH as well, thus impeding the progress of PC. The objectives of NPCP have not been achieved yet as the funding is lagging: *“I think most of our difficulties is money.” (O4T10, par. 86).* An interviewee expressed an uncertainty as to whether the MoH considers PC to be worth the effort, therefore, many hospices struggle. There is a lack of paid jobs in the PC sector. Physicians are expected to provide care for patients in their *"non-existent spare time" (D3J02, par. 24)*. The lack of generalist PC training leads to excessive demands and workload. The shortage of specialists is not expected to improve in the coming years. Training from abroad is not accredited, and the training centers for a new generation of PC providers are limited.

### Prospects and Goals of PC

Increasing awareness and understanding is a step to progress the field:

*[PC] It's evolving because the public is evolving, and it is understanding of what PC is. Evolving in their assumption of what they want, as far as their care. It's all really new here. (D3J02, par. 58).*

Most interviewees express their vision of making PC available to all patients and families in Israel. PC should be available in all stages of a disease. Israel should not be inferior to other countries in this respect. More jobs in the PC sector are needed to achieve this goal, at least a full-time PC physician and nurse in every Israeli hospital. PC should have the same status as other disciplines and be integrated into the culture of every hospital. Likewise, the outpatient services should contain PC. Interviewees mentioned that PC training must be on an equal footing with other medical fields so that doctors of all disciplines can use PC skills. Institutions that provide PC should be obliged to offer further training in the field. *The infrastructure for teaching, specialization, and management of PC professionals has improved significantly. For example, national PC courses have been developed for treatment teams. These are based on the Canadian* LEAP Model (Pereira et al., 2020) and will be mandatory for PC providers. An increase in the number of PC specialists comes with more key people in this field. Regular meetings to improve cooperation between experts have been launched. Various committees and national PC programs promote PC. The NPCP published a paper in Hebrew with recommendations for the further development of PC in Israel. The MoH has launched a pilot study based on this writing. Also, the newly founded Center for Dignified End of Life in Jerusalem supports PC research. The view on PC is changing, both in the MoH and in the general population.

## Discussion

This article describes the current situation of PC from the medical faculty members’ point of view. It provides a valuable insight into challenges that medical professionals face in their daily work. The study enables insights into personal, professional, and societal challenges.

In Israel, the flagship of PC is nurses. They are well-trained, act independently and provide the majority of PC services in Israel. Nurses coordinate the PC teams in both the outpatient and inpatient sectors. Nurses could be an important engine for PC, they are in direct contact with the patients, patients’ families, and in many cases have a good understanding of cultural context, which applies in particular to community nurses in rural areas (Silbermann et al., 2012). Palliative medicine is a new discipline in Israel. Since the MoH has defined the multidisciplinary PC teams (WHO, 2002), the cooperation between doctors, nurses, social workers and psychologists has been essential. The multidisciplinary team approach is positively perceived and emphasized despite financial difficulties, lack of human resources and trained professionals. The exploration demonstrated that the diversity in Israel places special demands on healthcare. Multiethnic, multilingual and spiritually diverse PC teams were emphasized, as helpful in dealing with patients and caregivers from different sociocultural and ethnic backgrounds because good communication and building relationships with patients and caregivers is particularly important in PC. Principles such as a holistic approach, cultural and religious coherence, and multidisciplinary approach, particularly respecting nurses’ competencies, are helpful in all medical disciplines and should not be applied only to PC. That would further improve the quality of care for all patients.

The Patient Rights Law requires that the patient be informed first and completely (Israel Ministry of Justice, 2018). Hence, physicians are to offer patients all information regarding their diagnosis and prognosis but let them decide for themselves how much they want to know (Blackhall et al., 1995; Shaulov et al., 2019). This law is extremely helpful in maintaining patient autonomy. In recent years, patients have been showing more interest and openness concerning their diagnosis and life expectancy. This openness simplifies doctor–patient communication. Patients want to know their prognosis, but this interest is bound to specific life events. Patients are more interested to talk about the quality of life and their individual live goals in the time remaining, than get an estimate of how much time they have left to live.

It has been suggested that it may be helpful to know some essential characteristics of different religious, ethnic or linguistic groups. It can help to improve patient care, avoid misunderstandings and reduce conflicts between caregivers and families (Shaulov et al., 2019). There are apparent differences between religious and ethnic groups, between secular and religious people, and between people from the rural and urban areas. However, feelings such as fear of death and dying are found equally among all people. Our study, similarly to an earlier study from the region (Abu Seir & Kharroubi, 2017), demonstrated that the basic principles of PC are independent of a patient’s ethnic background, religion or culture. Medical professionals involved in this study had very different wishes regarding their own dying. These preferences, however, should not affect patient care. In PC, each person has to be approached impartially addressing their values and beliefs regarding life and death. A clear plea for impartiality about a patient's religious, cultural, or ethnic background was expressed and the importance of collaboration across the borders stressed. The healthcare professionals must recognize that a person’s dignity and right to self-determination have to be acknowledged and prioritized. Besides addressing their own biases, attitudes, assumptions, stereotypes, prejudices, structures and characteristics that may affect the quality of care, healthcare professionals and their associated healthcare organizations should influence healthcare systems to reduce bias, also due to promoting equity within the workforce and working environment (Cherny, 2007).

The results underscore two interesting phenomena that may be unique to Israel. First, the results indicate that there is agreement that the principles of PC are independent of religion and culture and that Israeli health care medical faculties and health professionals are not very occupied with religious aspects of PC. It can be cautiously hypothesized that this is because Israel is a very diverse country in terms of religion and that religion plays an extremely important role in the daily life of the Israelis (independent of their religion) (Ben-Porat, 2017). Most of the Israelis are observant to some degree and religious elements are embedded in the Israeli healthcare system (Surbone et al., 2018). Hence attending to the religiousness of people with various religious degrees from different religions is a common practice (Halevy & Halevy, 2018). The second observation is that the results indicate that there is not much differentiation in the Israeli PC between religiosity and spirituality. This may also be a result of the deep religious Israeli culture. While PC in Israel may be very good in attending religious needs it may not be so good in attending to other spiritual needs that may or may not be religious by nature (e.g., finding meaning, sense of transcendence and connecting with something outside the self). The European White Paper strongly promotes the idea that spirituality is universal, deeply personal and individual and that spiritual care goes beyond formal notions of cultural beliefs or religious practice to encompass the unique capacity of each individual (Best et al. 2020). Emphasis should be on the idea that the path to spirituality is not always religious.

The provision of PC services in times of conflict is challenging and requires good planning and caution. If successful, it could contribute to understanding between the two sides (Curtis et al., 2019). Cooperation between conflicting parties in the field of PC can lead to relationship-building. Collaboration in one area contributes to peace and conflict resolution (Skinner & Sriharan, 2007). This study demonstrates that political conflicts do not affect patient care. The doctor–patient relationship between Palestinians and Israelis is unproblematic and professional. All patients, regardless of their origin, receive the same medical care. PC physicians are politically committed to peace in the region and enhance PC in the Gaza sector as crossing the borders have become more difficult. The linguistic barrier and certain distrust are acknowledged. In times of crisis, tensions between the patient groups are noticeable.

Israel is among the top quarter of the best served European countries in terms of specialized PC (Arias-Casais et al., 2020). The number of specialized PC facilities (1.4 per 100,000 inhabitants) is one of the best indicators of a country's level of PC development (Woitha et al., 2016). The lack of education and training opportunities has repeatedly been identified as one of the greatest obstacles to the development of PC (Centeno et al., 2017). As a result, many physicians find it difficult to think and talk about death and do not recognize the need for PC. In Israel, the infrastructure for teaching and specialization is improving. All medical schools (Elsner et al., 2021), three hospitals, and one ambulatory health service provide training in PC. A private company providing hospice services is approved as a venue for rotation for physicians during their PC training (IMA, 2022). There will soon be more PC professionals in medicine. The greatest hindrances to the further development of PC are the lack of funding. So far, PC is not yet included in the national health budget (Arias-Casais et al., 2020). Without sufficient financial resources, the goals set for PC cannot be achieved. Many physicians provide PC as an addition to their main duties, and therefore, do not have enough time for this important task. Despite these obstacles, PC in Israel is undergoing a change and therefore a good chance for a positive transformation in coming years. With research expanding and PC specialists discussing hindrances, goals, and solutions for the future of Israeli PC (Shaulov et al., 2019), PC services of good quality shall be available to all who can benefit from these.

### Limitations

Interviews were conducted in English, which was not the native language of either the interviewer or the interviewees. Conducting interviews in a participants own language may have enabled them to better articulate their thoughts, which may lead to semantic imprecisions. Controversial but heartfelt discussion about cultural and religion diversity in Israel may indicate that medical professionals merely shared their lay opinions, or are used to be flexible with all the diversity around. A hypothesis that needs further investigation.

## Conclusions

This article explored the status and characteristics of PC as seen by faculty members of the five medical faculties in Israel. PC is an integrative healthcare service that should be available to all patients, including children and their families at any stage of illness. Particular emphasis was on holistic care, multidisciplinary teamwork and a person-centered approach. PC principles apply regardless of ethnic, cultural and religious background. Patients have the right to discuss their diagnosis and prognosis as far as they wish. Despite high standards in some settings, PC does not currently reach all patients in Israel. The reasons are a lack of funding, educational opportunities and nationwide cooperation among experts. However, there are also many promising opportunities for positive development.

## Data Availability

The research data are confidential.

## The Global IMEP Initiative International Medical Education in Palliative Care–Research on Undergraduates’ Education Outcome–IMEP-RU-Israel

### Interview Guide

#### Key Persons of Palliative Medical Education in Israel

Name:_____________________________________.

Age:_____________________________________.

Gender:_____________________________________.

University: _____________________________________.

Faculty: _____________________________________.

First of all, I would like to thank you very much for agreeing to answer a few questions. It will take approximately 30 min for the whole interview. Is that ok for you?

Remember all that your responses and any information you provide will be treated as confidentially.

We developed this study for a better understanding of how prepared prospective doctors are to care for patients with chronic incurable disease.

## Your Person

- Which professional group do you belong to?
- Medical
- Nursing
- Psychology
- Other: _________________________________
- If you are a doctor, which department do you belong to?
- Anesthesiology
- Hemato-oncology
- Internal (other)
- Family medicine
- Palliative medicine
- Surgery
- Psychosomatic medicine
- Other: _____________________________________________________
- What kind of palliative medical education did you experience yourself?
- None
- Training as a core medical trainee (pre-qualification)
- Train the trainer course
- Master Palliative Care/Medicine
- Cardiff palliative diploma
- Other: _____________________________________________________
- Which is your role in (palliative medical) education at your faculty?
- Dean
- Associate Professor
- Professor and Chair
- Other: _____________________________________________________

## Personal Understanding of Palliative Care

- Please describe your own definition of palliative care.
- In which way do you think is palliative care different to the treatment in other departments?

## Experiences with Palliative Medical Education

- Please describe the quality of palliative medical education at your faculty.
- In your experience, do students feel better prepared for the care of incurable and dying patients after having attended your courses?
- Please describe the quality of palliative medical education in the whole country of Israel.

## Country-Specific Aspects of Palliative Care in Israel

- In Israel, is it more common to inform the patient or his relatives about his medical situation? What do you think about this personally?
- Is it common for patients to ask for prognosis? What do you think about this personally?
- What aspects have to be considered in palliative care/medicine concerning the variety of religions in Israel?
- How do you think does the political situation of Israel influence palliative care?

## Working in a Multidisciplinary Team

- Please describe the role of a multidisciplinary team in palliative care.
- Please describe the special role of palliative care nurses in Israel.

## Personal Attitude Toward Death and Dying

- Please, describe your attitude toward death and dying
  - As a health care professional
  - As an individual person

## Role Concept and Teaching Aspects:

- How do you see yourself as a doctor?
- Please describe your role as a teacher.
- Please describe aspects which you consider crucial when teaching palliative care.
- Please describe main competencies which a student should learn.

## Personal Support for Students and Doctors

- Please describe the kind of support for students or doctors working with incurable and dying patients.

## Future

- What are your hopes for future palliative medicine in Israel?
- What are your hopes for future palliative medical education in Israel?

Thank you very much! You have contributed to a better understanding of palliative care/medicine education in Israel, especially at your university.

Do you have any further questions?

Would you like to get the results of the study? Then please leave your email address with me.
